# Comparing breastfeeding experiences between mothers spending the traditional Chinese confinement period in a confinement centre and those staying at home: a cohort study

**DOI:** 10.1186/s13006-020-00353-1

**Published:** 2021-01-06

**Authors:** Siew Cheng Foong, May Loong Tan, Wai Cheng Foong, Jacqueline J. Ho, Fairuz Fadzilah Rahim

**Affiliations:** RCSI & UCD Malaysia Campus, 4 Jalan Sepoy Lines, George Town, Penang Malaysia

**Keywords:** confinement centre, postpartum care, breastfeeding, support, barriers, Chinese

## Abstract

**Background:**

Ethnic Chinese mothers in Malaysia adhere to 30 days of traditional postpartum practices (the “confinement period”) aimed at recuperation after delivery. Recently there has been an emergence of confinement centres (CCs) where mothers stay and receive traditional confinement care. Ethnic Chinese mothers have low breastfeeding rates. There are concerns that practices in CCs could contribute to this but no data exists. We described mothers’ breastfeeding experiences at CCs and identified areas for potential improvement in breastfeeding support.

**Methods:**

Ethnic Chinese mothers intending to breastfeed their healthy infants were recruited post-delivery between August and October 2017 then, at 1 and 6 months, they were telephone interviewed about their experience. For every participant going to a CC after the birth, another mother going home (“home”) for her confinement was recruited. Chi-square test was used to compare groups and multiple logistic regression was used to assess the effect of confinement place on exclusive breastfeeding.

**Results:**

Of 187 mothers, 88 (47%) went to CCs. Significantly more were primipara and fewer had previous breastfeeding experience. Response rates for the 1- and 6- month interviews were 88% (CC) versus 97% (home); and 77% (CC) versus 87% (home) respectively.

Exclusive breastfeeding rates were similar between the groups: 62% (CC) versus 56% (home) at 1 month (*p* = 0.4); and 37% (CC) versus 42% (home) at 6 months (*p* = 0.5). Multiple logistic regression did not show that CCs were a factor affecting exclusive breastfeeding rates at 1 month, (adjusted odds ratio [aOR] 1.7, 95% confidence interval [CI] 0.9, 3.3), or 6 months (aOR 0.9, 95% CI 0.4, 1.7). However, significantly more CC participants only fed expressed breast milk. Despite 66% of CC participants reporting that their centre supported breastfeeding, only 6 (8%) CC participants compared to 66 (69%) of home participants roomed-in with their baby (*p* < 0.001). The proportion encountering breastfeeding difficulties were similar between groups. CC participants sought help for breastfeeding problems mainly from CC staff and support groups while home participants obtained help from friends and healthcare professionals.

**Conclusions:**

Breastfeeding rates appeared to be similar at CCs and at home during the confinement period, but there were gaps in how CCs supported breastfeeding. Targeted training to CC staff to support breastfeeding may result in better outcomes for mothers staying in CCs.

## Background

Breastfeeding is the cornerstone of infant nutrition and the dangers of not breastfeeding are well recognised [1]. The early initiation of breastfeeding after birth and subsequent early postpartum establishment of breastfeeding are important for successful breastfeeding [2]. Close and continuous proximity of mother and baby 24 h a day is also important to the establishment of breastfeeding [3]. In some traditional cultural postpartum practices, restrictions to contact between mother and baby may occur. These may affect mother-infant interaction and breastfeeding practice.

Malaysia has a multi-ethnic society and each ethnic group has its own traditional postnatal practice. Chinese ethnicity is one of the three major groups and despite modernisation, most Chinese women adhere strictly to a 30-day traditional postpartum period, known locally as the “confinement period” or “zuo yue zi” [4–7].

These practices are based on the traditional Chinese belief system about the maintenance of Yin and Yang in the mother after childbirth so that her health is restored. Many believe that non-adherence to these practices will result in potentially long-term adverse effects on the quality of life to the mother. Influences of relatives, in particular the new mother’s mother, her mother-in-law and her grandmother, are factors that ensured that these practices are carried out and passed down from generation to generation [4, 7].

During the confinement period, the mother would be assisted fully at her home by someone (or sometimes more than one person), ensuring that she gets enough rest, providing the appropriate diet, caring for the newborn baby and ensuring she abides by confinement practices such as keeping warm [5]. This usually means the mother avoids draughts or taking showers for the whole month [4, 7, 8]. This person was traditionally a close female relative [6] but practices have evolved to hiring a “confinement lady” (yue so) who is considered an expert in the necessary diet and practices [8, 9]. The confinement lady would be employed to stay in the new mother’s home after delivery for at least 4 weeks [9].

Over the last decade, a new method of providing this care emerged and is increasingly replacing the role of confinement ladies or female relatives at home. Confinement centres (CCs) provide a place for postpartum Chinese mothers to stay during their confinement period and observe traditional postpartum practices under the care of the CC staff [9]. A typical CC is often a converted house with several rooms for mothers and a room designed as a nursery where the babies are all placed. Mothers may share rooms and typically all the babies would be in a single ‘nursery’. This centre would be staffed by women who have experience with newborns and are familiar with the Chinese cultural confinement requirements, a cook to prepare the required confinement diet for mothers and occasionally qualified nurses to look after the well-being of mothers and babies [9]. Although care of the infant is in no way neglected because the focus of these CCs is mainly towards providing traditional care for the mother, compromises in mother-infant bonding and breastfeeding may occur. This has led to concerns that breastfeeding may not be adequately supported in CCs and further compromising breastfeeding rates for the Chinese population, which is currently the lowest among the major ethnic groups in Malaysia [10, 11], despite evidence that a high proportion of them intend to breastfeed [10].

There are currently no published data about breastfeeding practices in confinement centres and this is important because the development of such centres is a growing phenomenon across Asia and beyond. The aim of the study is to describe the breastfeeding experience of ethnic Chinese mothers who stayed in CCs during the traditional confinement period and compare the breastfeeding outcomes at 1 and 6 months with those who stayed at home, to determine if the place of confinement had any effect on breastfeeding, identify current practices and areas for potential improvement in breastfeeding support in confinement centres.

## Methods

This was a prospective cohort study. Participants were recruited from the postnatal wards of six hospitals in Penang, Malaysia between August and October 2017. Malaysian mothers of Chinese ethnicity with the intention to breastfeed their healthy term infants were recruited post-delivery, then telephone interviewed about their experience at 1 and 6 months later. We excluded single mothers because they may have different social support which may affect breastfeeding. Recruitment was done by the infant’s attending doctor, who apart from this was not otherwise involved in the study. Women who chose to go to a CC were recruited on a consecutive basis and the control selected was as far as possible the next woman from the same hospital who planned home confinement. Participants were told that we wanted to study their breastfeeding experience and the information would be used to improve mothers’ experiences in the future. Written consent was obtained from the participants prior to the commencement of study.

After consent was obtained, maternal and infant baseline characteristics were collected before discharge. These included maternal age, educational level, occupation, parity, previous breastfeeding experience, place of intended confinement, infant’s date of birth, hospital of birth, gender, gestational age at birth, mode of delivery and birthweight. After discharge, there was no contact between the research team and the participant until after her 30-day confinement period.

A telephone interview with the participant was conducted 1 month postpartum (the end of the confinement period). The focus of this telephone interview was to seek the breastfeeding experiences of participants who stayed at CCs and compare it with those at home. The questions included infant’s feeding practices, opportunity to room-in and/or spend time with their infants, problems encountered during breastfeeding (such as engorgement, mastitis, insufficient milk), sources of help for these problems and any perceived barriers to breastfeeding. Participants were given the opportunity to elaborate on any of the questions asked. They were also asked at the end of the interview if there was anything positive or negative about her confinement centre that she would like to share with us. The second telephone interview to collect data on breastfeeding practices was conducted when the infant was 6 months old. All questions used had been tested in a separate group of breastfeeding mothers not involved in the study. Both telephone interviews were conducted by three trained research staff and the responses were directly entered into a specially designed interview form.

### Sample size calculation

Sample size was calculated to be 94 in each group taking into consideration a 20% drop out rate. This was based on the National Health and Morbidity Survey 2016 exclusive breastfeeding prevalence for Malaysian Chinese mothers at 0 to 2 months of 42.5% [12] and our hypothesis that there would be a 50% lower exclusive breastfeeding rate in the CC group compared to the home group.

### Breastfeeding definitions

We defined exclusive breastfeeding according to the World Health Organisation’s definition which means no other food or drink, not even water, except for medicines, vitamins and breast milk (including breastfeeding, milk expressed or from a donor). Any breastfeeding was defined as breast milk (including breastfeeding, milk expressed or milk from a donor), and any other food or liquid including non-human milk and formula [13].

Data on breastfeeding was collected during the 1- and 6- month interview asking the mother how she was mainly feeding her baby.

### Data analyses

We tabulated the baseline demographics of the mothers according to place of confinement. Continuous data was presented as mean with standard deviation (SD) and categorical data presented as frequency with percentage (%). Chi-square analysis was used to compare the baseline characteristics between participants staying in confinement centres (CCs) and those staying at home. Comments from free field options were tabulated and categorized into groups. Some of these free field responses were quoted as illustrations. Simple logistic regression was used to determine whether CCs affected breastfeeding rates (any breastfeeding and exclusive breastfeeding) at 1 month and 6 months postpartum and presented as crude odds ratio (OR) with 95% confidence interval (CI). We modeled the likelihood of exclusive breastfeeding or any breastfeeding as a function of CCs using multiple logistic regression by adjusting for other clinically important variables. The results were presented as adjusted odds ratio (aOR) with 95% CI. Statistical analysis was done using Stata13 [14]. We considered a *p* value of less than 0.05 as significant.

## Results

A total of 187 mothers consented to participate, of which 88 (47%) chose to stay in a CC. Of the 99 (53%) participants who chose to go home during their confinement period, a third of them employed confinement ladies to provide confinement care at home, while the remainder received their care from family members.

**Fig. 1:**
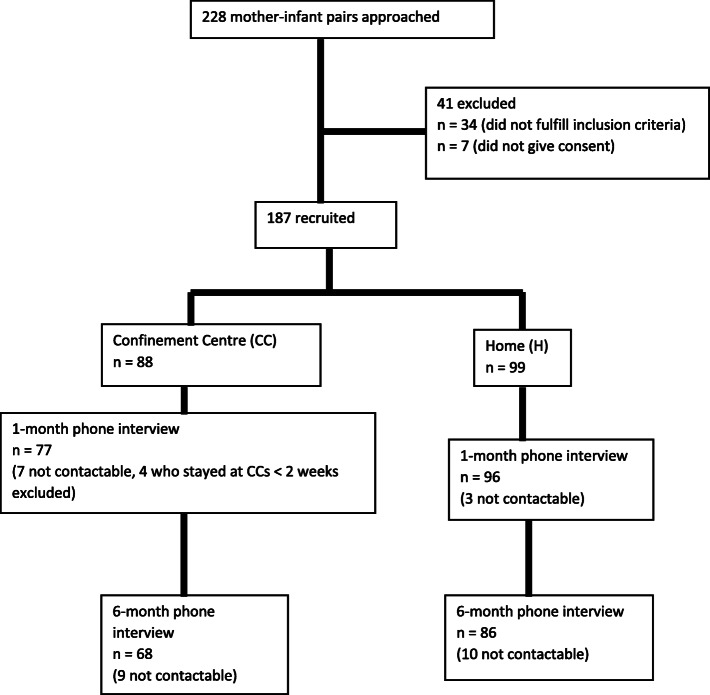
Study flow diagram

We were able to interview 77 (88%) participants from the CC group and 96 (97%) from the home group at 1 month postpartum. At 6 months, 68 (77%) from the CC group and 86 (87%) from the home group completed the interview. (See Fig. 1).

### Baseline characteristics

The maternal and infant baseline characteristics are presented in Table 1. The overall mean maternal age was 32.3 (SD 4.0) years with 54% having previous experience with breastfeeding and 57% delivering in a Baby Friendly Hospital Initiative (BFHI) accredited hospital. Most mothers had a tertiary education and all had at least secondary school education, which reflects what is expected in Penang. The overall mean infant gestational age was 38.7 (SD 1) weeks and mean birthweight of 3149 (SD 322) g. There were no differences in the age, education background, delivery at a BFHI accredited hospital, mode of delivery; infant gestation and birthweight between the two groups. However, we found that significantly more primiparas (53% CC vs 34% H, *p* = 0.01) and fewer mothers with prior breastfeeding experience going to CCs (44% CC vs 63% H, *p* = 0.01).

**Table 1:** Baseline characteristics of the mothers and infants (*N* = 187)

Characteristics	Place of confinement, ***n*** (%)	
	Confinement centre (*n* = 88)	Home (*n* = 99)
Age of mothers (years), mean (SD)	32 (4.3)	32 (3.3)
Received tertiary education	70 (79.6)	80 (80.8)
Delivery in BFHI hospital	49 (55.7)	57 (57.6)
Primipara*	47 (53.4)	34 (34.3)
Had previous breastfeeding experience*	39 (44.3)	62 (62.6)
Mode of delivery		
Vaginal	41 (46.6)	51 (51.5)
Instrumental	15 (17.1)	16 (16.2)
Caesarean	32 (36.4)	32 (32.3)
Male infant	45 (51.1)	56 (56.6)
Gestational age at birth (weeks), mean (SD)	38.72 (1)	38.67 (1)
Infant’s birthweight (g), mean (SD)	3141.25 (303.6)	3156.07 (339.2)

* *p* value < 0.05

### Breastfeeding practices at 1 month and 6 months

At 1 month postpartum, all the interviewed participants were continuing to breastfeed, except two mothers from the home group. There was no difference in the exclusive breastfeeding rates between the two groups (62% for CC and 56% for home, *p* = 0.36). Similarly, at 6 months, there was no difference in the exclusive breastfeeding rates between the two groups (37% for CC and 42% for home, *p* = 0.70). Simple logistic regression showed no association between exclusive breastfeeding rates and place of confinement at 1 month, (OR 1.3, 95% CI 0.7, 2.4) or at 6 months, (OR 0.8, 95% CI 0.4, 1.6). Multiple logistic regression adjusted for known clinically important confounders (education level, past breastfeeding experience, place of delivery, mode of delivery, spent more than 6 h a day with baby, roomed-in with baby at night, employment status at 6 months) also did not show that the CC or home was a factor affecting exclusive breastfeeding rates at 1 month (aOR 1.7, 95% CI 0.9, 3.3) and at 6 months (aOR 0.9, 95% CI 0.4, 1.7) or ‘any breastfeeding rates’ at 6 months (aOR 1.1, 95% CI 0.5, 2.2). We were unable to estimate the odds ratio for ‘any breastfeeding rates’ at 1 month because all except two participants from the home group were breastfeeding (see Table 2).

**Table 2:** Crude and adjusted ORs for breastfeeding at 1 and 6 months defined by place of confinement

	Odds Ratio, OR (95% CI)	***p*** value	Adjusted Odds Ratio, aOR (95% CI)^a^	***p*** value
At 1 month				
Exclusive breastfeeding	1.3 (0.7, 2.4)	0.42	1.7 (0.9, 3.3)	0.14
Any breastfeeding^b^	Not estimable		Not estimable	
At 6 months				
Exclusive breastfeeding	0.8 (0.4, 1.6)	0.52	0.9 (0.4, 1.7)	0.67
Any breastfeeding	1.0 (0.5, 1.9)	0.99	1.1 (0.5, 2.2)	0.82

^a^Adjusted for maternal education level, past breastfeeding experience, place of delivery, mode of delivery, spent more than 6 h a day with baby, rooming-in at night, employment status at 6 months

^b^All mothers except 2 from the home group were practicing some form of breastfeeding

Among all participants who were continuing to breastfeed at 1 month (*n* = 171), 29% were breastfeeding directly from the breast, 49% only fed their infants with expressed breast milk (baby was never latched to breast) and 21% used a combination of both. Significantly more participants from CCs were only feeding expressed breast milk compared to those from home (62% vs 39%, *p* < 0.001) at 1 month (See Table 3).

**Table 3:** Methods of breastfeeding by place of confinement at 1 month

	Confinement centre (***N*** = 77) ***n*** (%)	Home (***N*** = 96) ***n*** (%)
Expressed milk feeding only	48 (62)	37 (39)
Direct latch feeding only	9 (12)	41 (43)
Direct latch and expressed milk feeding	20 (26)	16 (17)
Stopped breastfeeding	0	2 (2)

Among participants who only fed expressed breast milk at 1 month (*n* = 85), 41% of them stopped breastfeeding by 6 months compared with 35% of those who had included direct latching to their breastfeeding routine (*p* = 0.64). We explored reasons for not directly breastfeeding at 1 month. The main reason was the perception that direct breastfeeding was very time consuming and prevented them from resting properly (*n* = 28). Other reasons included having problems associated with latching (*n* = 22), the need to monitor baby’s milk intake (*n* = 14), and difficulty accessing their baby in the nursery or being discouraged from directly latching their infants (*n* = 8). One mother did not want to place the baby near her breasts saying “I feel dirty because I haven’t had a shower for so long.”

### Breastfeeding experience

Overall, 66% of the participants in the CC group reported that their centre was supportive of breastfeeding. When asked reasons for saying so, they cited one or more reasons which we categorised into the following: staff helped them overcome breastfeeding problems, mental support from staff, breastfeeding education given by staff (especially for mothers with no prior breastfeeding experience), availability of peer support from other mothers in the same centre. Of note, none of the participants stated that they perceived their centre to be supportive of breastfeeding because they had received encouragement to spend time with their infant or to breastfeed at night.

For both groups, we asked if they faced any hindrance to breastfeeding. Ten participants from the CC group reported that they did encounter one or more forms of hindrance which we categorized into the following: staff were not helpful with breastfeeding problems; staff encouraged formula feeding; mother was asked to pump less breast-milk because there was inadequate refrigerator storage space; family members were against breastfeeding; inappropriate information given regarding breastfeeding such as the need to stop breastfeeding for a jaundiced baby or for diarrhea or skin rashes; or misconceptions such as “baby would be hungry without formula”, “breast milk will cause indigestion”, “breastfeeding was troublesome and would result in a clingy baby”. However, not being allowed to breastfeed at night was not cited as a perceived hinderance to breastfeeding. Interestingly, three of the participants who said they had some hindrance to breastfeeding had earlier considered their centres to be supportive of breastfeeding. On the other hand, eight participants from the home group indicated that they had faced breastfeeding hindrance either because their family members or hired confinement lady did not support breastfeeding.

### Sleeping arrangements during the confinement period

Despite 66% of CC participants reporting that their centre supported breastfeeding, only 57% reported that their CC allowed mothers to share a room with their babies. However, even among the 43 participants whose CCs allowed them to room-in with their babies, 38 participants said they chose not to do so. They would sleep in their own room (sometimes with a few other mothers in the same room) while their babies slept in a nursery. When we compared CC participants with participants at home, only 8% of CC participants actually slept with their babies while 69% of those from home did so (*p* < 0.001).

Only 73% of CC participants reported that they could access their babies at any time of the day or night, while the remaining 27% had some restrictions. In particular, 17 participants reported that their CC discouraged or disallowed breastfeeding at night. Other restrictions included designated times to be with or see their baby through a window in the nursery. We illustrated how much access a mother has to her baby if they did not room-in together in Table 4.

**Table 4:** Mother’s access to baby in the nursery (for participants who did not share a room with their babies, *n* = 71)

Description	***n*** (%)
Baby could be brought out of the nursery at any time to be with mother. In addition, mothers could see their babies from outside of the nursery through a window any time of the day or night.	51 (66)
Baby could be brought out of the nursery at any time to be with mother. Window for mothers to see their babies in the nursery only open for certain hours of the day.	8 (10)
Baby could be brought out of the nursery at any time to be with mother but unable to see baby while in the nursery.	2 (3)
Baby only allowed out of the nursery during the day. No physical access to baby at night. However, mothers can see their babies through a window from outside of the nursery any time of the day or night.	5 (6)
Baby only allowed out of the nursery during the day. No physical access to baby at night. Window for mothers to see their babies in the nursery only open certain hours of the day.	3 (4)
Baby only allowed out of the nursery during the day. No physical access to baby at night. Unable to see baby from outside of the nursery.	2 (3)

### Breastfeeding problems encountered and sources of help

Breastfeeding problems were experienced by 62 participants in the CC group (81%) and 73 participants in the home group (76%). Among those with breastfeeding problems, significantly more participants in CCs (79%) compared with those at home (59%) encountered latching difficulties, breast engorgement, blocked ducts, mastitis, abscess and sore nipples (*n* = 49 vs 43, *p* = 0.03). However, there was weak evidence that inadequate milk production was more commonly reported in the CC group compared to the home group (*n* = 40 vs 33, *p* = 0.06).

The number of participants who perceived that family members were the main source of hindrance to breastfeeding was similar in both groups (*n* = 9 vs 8, *p* = 0.64).

Sources of help for breastfeeding problems were different for participants in CCs and home. Participants in CCs mainly sought help from centre staff (*n* = 30, 39%) and breastfeeding support groups (*n* = 15, 19%) while most home participants obtained help either from friends (*n* = 16, 17%) or healthcare professionals (*n* = 19, 20%). Only 7 (7%) of participants staying at home obtained help from family members. Of the 32 participants who hired a ‘confinement lady’, eight sought their assistance. From both the CC and Home groups, only 7 participants (4%) sought help from lactation consultants.

## Discussion

We found that exclusive breastfeeding rates at 1 month and 6 months appeared to be similar for mothers who went to CCs and those who stayed at home. This is contrary to current perceptions that CCs are a cause of early breastfeeding cessation due to numerous practices in CCs that hinder breastfeeding. One such practice which we found in our study was that CCs discouraged mothers from rooming-in with their babies. This was worrying as it causes significant separation of mother and infant and could disrupt the establishment as well as continuation of breastfeeding [15]. The practice of separating mothers from their babies at CCs was most likely due to the cultural emphasis on the need for postnatal mothers to rest during the confinement period [4, 7, 8]. A mother who is separated from her baby would not be able to detect the baby’s feeding cues and thus, may miss breastfeeding opportunities. This turn may result in lower milk production [3].

It was interesting to note that more mothers were feeding expressed breast milk to their babies at CCs compared to home. While we did not further explore reasons for this, the most likely reason was that these mothers had the intention to breastfeed. However, due to limitations in access to their infants, both due to lack of rooming-in and in some cases restrictions in access to nurseries, they had chosen to do this in order to sustain breast milk feeding. We do not know if there was also a possibility of influences from commercial breast-pump industries [16] or if women in CCs could also be influencing each other to express milk rather than to directly breastfeed. There were also more primipara mothers in CCs, and a study in Hong Kong found that mothers with no previous breastfeeding experience were more likely to exclusively feed expressed milk [17].

While we do not have data for Malaysia, there are reports that expressed milk feeding is an increasing trend among ethnic Chinese mothers in neighbouring Singapore and Hong Kong [17–19]. This is a concern because there is a growing body of evidence that feeding expressed breast milk might be different from feeding at the breast. Studies have found that there is higher risk of asthma [20] and childhood obesity [21–23] with expressed breast milk feeding compared to direct breastfeeding. Studies have also found that only feeding expressed breast milk could result in early cessation of breastfeeding [17, 23–25]. Therefore, it is important for future studies to explore this trend to understand and address the factors that influence the choice to feed expressed milk rather than directly breastfeed.

Given all the above barriers to breastfeeding in CCs, it had been a surprise to find there were no difference in exclusive breastfeeding rates at 1 month and 6 months. A possible reason could be because all mothers included in this study had the intention to breastfeed and having such an intention is a recognized factor affecting breastfeeding duration [26]. We believe that this intention was the main factor why mothers in this study persevered with breastfeeding despite presence of some practices in CCs that hindered breastfeeding. Another reason for this finding was the perceived support that the mothers had from their CCs, even though there were gaps in the provision of support and a lack of understanding of what appropriate breastfeeding support actually entails. For example, despite restrictions in access to their babies and being discouraged from rooming-in or feeding at night (both of which are known barriers to breastfeeding), these mothers felt that their CC was supportive of breastfeeding. There is evidence that mothers who perceive that they are supported are more likely to successfully breastfeed [3].

Apart from the above, the breastfeeding experience in the two settings was similar. Mothers from both groups encountered people who hindered breastfeeding; some mothers in CCs had CC staff who discouraged breastfeeding while some of those who stayed at home had hired confinement ladies who discouraged breastfeeding. In both the groups, family members were also cited as a barrier to breastfeeding.

We found that there were more first-time mothers in the CC group. While there are no known published reports on this, the most likely reason is because CCs are a relatively new phenomena and are becoming increasingly available while the traditional confinement is less available due to the changing social structure, living arrangements and reduced availability of the traditional confinement lady. Younger mothers are more likely to use this facility while more experienced mothers might opt for what they had before, i.e. home care. In addition, first time mothers may also lack confidence to look after the baby themselves at home without a ‘traditional confinement lady.’ Another reason might be that multiparous mothers did not want to be separated from their other children. Future studies could be done to explore the validity of these explanations.

Our study is important because over the past decade there has been a rapid expansion of CC services not only in Penang and throughout Malaysia but also in other parts of the world including Singapore, Hong Kong and mainland China. Similar centres are also opening to cater for the traditional needs of the other Malaysian ethnic groups. Therefore, this study offers insight into the level of breastfeeding support that is currently being offered and the findings will be useful in determining how support can be offered to these centres and to mothers at home. We noted that only a few mothers sought help from lactation consultants or peer support groups. There is currently no formal recognition of lactation consultants in Malaysia and the number of certified consultants is very low. Peer support groups exist but again our data showed that these were not sought either by the CC or home participants. Efforts to link CCs and mothers at home with these groups are needed. This study also provides insight into the training needs of the CC staff.

A limitation of our study would be that our findings reflect only the views of mothers who at recruitment had the intention to breastfeed. These findings do not apply to mothers without an intention to breastfeed, and such studies are needed. In addition, this study is limited because we did not have data on which CC the mothers went to. However, while we believe that our sample of mothers reasonably represents the mothers using CCs in Penang, we do not believe that we have a reasonable representation of the CCs in Penang. It is likely not all CCs in Penang were represented in the data and it is possible that several of our participants could have been at any one of the CCs. Practices in each CC were different, with some more supportive of breastfeeding than others. Given that we recruited women who intended to breastfeed, we may have inadvertently selected CCs that were more likely to be supportive of breastfeeding. Therefore, our results might not be generalizable to all mothers attending all CCs in Penang. Another limitation of the study was the sample size. We based our sample size on the assumption that we could expect a 50% lower exclusive breastfeeding rate in the CC group. There were no available data to guide us in this estimate and we now believe this may have been an overestimation of the expected difference. Indeed, the sample size was barely adequate to meet this 50% assumption. Therefore, there is a possibility that there could be a difference between the two groups that we failed to demonstrate due to this overestimation.

## Conclusions

We found that spending the traditional confinement period in CCs or at home did not appear to result in a difference in both exclusive breastfeeding and any breastfeeding rates at one and 6 months postpartum among Chinese mothers who intended to breastfeed. However, when describing their experience, we found definite gaps in how CCs were supporting breastfeeding. Ways to overcome this need to be explored. One such intervention might be to provide training to improve CC staff’s ability to provide breastfeeding support. Such training would need to be tailored specifically to their needs and thus CC staff input into the design of an educational training package is recommended. Future studies could also be done on all mothers regardless of their intention to breastfeed.

## Data Availability

The datasets used and/or analysed during the current study are available from the corresponding author on reasonable request.

## Abbreviations

BFHI: Baby Friendly Hospital Initiative
CC: confinement centre
vs: versus
